# Pulmonary blood flow quantification in humans from 15O-water PET

**DOI:** 10.1007/s12149-025-02035-6

**Published:** 2025-03-14

**Authors:** Oona Rainio, Henri Kärpijoki, Juhani Knuuti, Riku Klén

**Affiliations:** Turku PET Centre, University of Turku and Turku University Hospital, Turku, Finland

**Keywords:** 15O-water positron emission tomography, Compartmental modelling, Dynamic positron emission tomography, Pulmonary blood flow

## Abstract

**Purpose:**

Dynamic positron emission tomography (PET) imaging has commonly been applied to study blood perfusion in the human brain and heart, but there is a very limited amount of existing research about the suitability of this method for many other organs of interest. Here, we focus on the quantification of pulmonary blood flow (PBF) in human lungs. We evaluate both the potential of the $^{15}$O-water PET imaging via compartmental modeling with automatic volume of interest (VOI) selection for PBF quantification and study the possible differences in PBF caused by different patient characteristics such as age or sex.

**Procedures:**

We systematically fit the one-tissue compartment model to the mean time-activity curves derived from the $^{15}$O-water PET data of 103 patients. The machine learning-based segmentation tool TotalSegmentator is utilized to find segmentation masks for different lung lobes and right ventricle of the heart. Additionally, we automatically remove the majority of the air inside the lung lobe VOIs and the areas surrounding subclavian arteries and brachiocephalic veins with the help of binary erosion and dilatation operations. After the model fitting, we evaluate possible differences in the results caused by age, sex, weight, and body mass index (BMI) by performing Mann–Whitney *U* tests between different patient subgroups and computing Spearman’s correlations coefficients.

**Results:**

The estimated PBF within all the lung lobes had a mean of1.21±0.825 mL/min/cm$^3$ and a median of 1.03 mL/min/cm$^3$, but this value was notably lower in right lower lung lobe and much higher in the upper lung lobes. The PBF was higher in both the female patients and in the patients under 65 years but not statistically significantly so. The individual variation was very high.

**Conclusions:**

The PBF quantification based on $^{15}$O-water PET imaging combined with our automatic VOI selection method is an effective method to produce relatively realistic results. In case of upper lung lobes, the results are likely overestimated if pulmonary vessels are not removed from the VOI. The accurate estimation of the air volume within the lung lobe VOIs is also a non-trivial problem. More research on this topic is warranted to find whether there is a decreasing trend between PBF and age or significant differences between the sexes.

## Introduction

Dynamic positron emission tomography (PET) can be applied to derive accurate kinetic information on blood perfusion, oxygen extraction, metabolism, and other body functions [1]. PET imaging utilizes short-lived radioactive tracers whose movements in the body are recorded based on the gamma radiation created during the radioactive decay process [2]. After a dynamic PET scan, the resulting data is presented as a series of three-dimensional (3D) images that show the tracer concentration in the body at different moments of time.

In a dynamic PET image, each voxel in the 3D space has a time-activity curve (TAC) that shows the tracer concentration at that point as a function of time. In order to estimate perfusion values from the TACs, we often use compartmental modelling [3]. This method is based on the idea that the changes in the tracer concentration within the different tissue types or organs can be described by a set of differential equations defining how the tracer is exchanged between a certain number of compartments. For instance, the standard method of cerebral blood flow estimation is to compute the rate constant of the $^{15}$O-labelled water delivery from the arterial blood to the brain tissue [4].

However, there is very little PET research about pulmonary perfusion of humans [5]. This is partially because of the limitations caused by the short axial field of view of older PET cameras but also due to the unique challenges of the pulmonary anatomy. Whereas most of the tracer concentration in the other organs is delivered from the arterial blood during the systemic circulation, the bronchial circulation of the lungs is very low compared to the pulmonary blood flow. Thus, the input function in the compartmental model needs to be the tracer concentration in the pulmonary blood estimated from the right ventricular cavity of heart instead of the descending or the ascending aorta [6] and this input is more difficult to compute accurately because of cardiac motion [7]. Additionally, the position of the lower lung lobes significantly varies due to respiratory motion.

We found only two studies related to pulmonary blood flow (PBF) quantification in humans based on $^{15}$O-water PET imaging: In 1995, Schuster et al. [8] estimated PBF in 15 healthy subjects and five cardiomyopathy patients, observing high correlation between the results obtained with $^{15}$O-water PET and $^{68}$Ga-gallium-albumin macroaggregates. In 2017, Matsunaga et al. [9] investigated the impact of adding an additional perfusable tissue parameter to the model introduced by Schuster et al. while computing PBFs of nine lung cancer patients.

In this paper, we explore the potential of the $^{15}$O-water PET imaging for the PBF quantification further. Combined with a new automatic total-body segmentation tool TotalSegmentator introduced by Wasserthal et al. [10], we derive TACs from over hundred patients and systematically fit the same compartment model as in earlier research [8, 9] for all of them. We also study whether there are significant differences in the PBF estimates caused by sex, age, weight, or body mass index (BMI) of the patients.

## Materials and methods

### Software

The anatomic structures of interest were located in the PET images according to the automatic segmentation of computed tomography (CT) images by TotalSegmentator (version: 2.0) [10]. The PET and CT images and TotalSegmentator masks were visually checked with Carimas (version: 2.10) [11]. Image analysis and TAC extraction was performed with Python (version: 3.9.9) [12], and the model fitting and statistical testing was performed with R (version: 3.4.1) [13].

### Data and pre-processing

The PET/CT data were retrospectively collected from 103 patients referred for total-body PET perfusion imaging at Turku PET Centre in Turku, Finland, between August 2022 and January 2024 due to a suspected coronary artery disease. Prior the PET imaging, they had been imaged with coronary CT angiography based on which they could not be cleared of a diagnosis of an obstructive disease. The mean age of the patients was 64.8 with standard deviation of 9.33 years (interval: 30–83 years), and the male–female sex ratio was 47:56.

The imaging was performed with Biograph Vision Quadra (Siemens Healthineers) PET/CT scanner which has an axial field of view of 106 cm. During the imaging, the patients lay supine on a bed. A target dose of $^{15}$O-water (350 MBq ±10%) was injected as an intravenous bolus over 15 s (Radiowater Generator, Hidex Oy, Finland). The measured activity of the dose varied from 297 to 408 MBq. The image acquisition was started 30 after the start of the tracer bolus. The PET images were reconstructed with ordered-subsets expectation maximization (OSEM) algorithm with a point-spread function and time of flight using 3 iterations and 5 subsets (PSF+TOF 3i5s). They were corrected for decay with a decay factor of 1.30906, randoms via the delayed event subtraction method, attenuation via a measured attention correction with CT information, and scatter via model-based with relative scatter scaling with a scatter factor of 0.409329. The final dynamic PET images consisted of $220\times 220\times 380\times 24$ image points with a voxel size of $1.65\times 1.65\times 3.00$ mm$^3$ and time intervals of 14$\,\times \,$5 s, 3$\,\times \,$10 s, 3$\,\times \,$20 s, and 4$\,\times \,$30 s. The total-body CT images contained $512\times 512\times 380$ voxels.

**Fig. 1:**
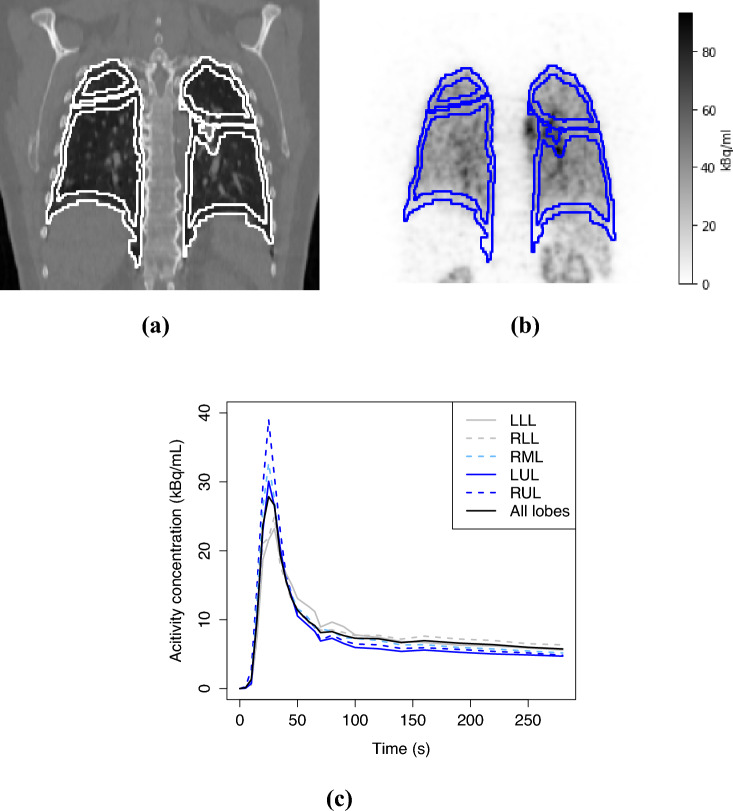
The outlines of the final hollow VOIs of four lung lobes (RML not visible here) in one cropped coronal slice of **a** the original CT image and **b** the PET image, and **c** the mean TACs of the lung lobes from this patient

The volume of interest (VOI) was defined for left lower lobe (LLL), right lower lobe (RLL), right middle lobe (RML), left upper lobe (LUL), and right upper lobe (RUL) of the lungs, and the right ventricle (RV) of the heart were created in a fully automatic way. First, we derived the initial VOIs according to the TotalSegmentator masks obtained for the CT images. To remove the air inside the lungs from the lung lobe VOIs, we removed the interior more than three adjacent voxels away from the surface of the TotalSegmentator VOIs. This was done automatically with the help of the binary erosion function. Additionally, we removed the area surrounding the right and the left subclavian arteries and the right and the left brachiocephalic veins from the lung VOIs by using TotalSegmentator masks of these four vessels enlarged with binary dilation to avoid spill-over effect. Finally, we only included the greatest connected components of the VOIs for both the five lung lobes and the RV, to avoid the potential false positive segmentation outside these anatomic structures. The resulting VOIs are robust, but this is taken into account later while defining the model. Based on these VOIs, we computed the mean TACs for each lung lobe separately and over all the five lung lobes and then used linear interpolation to obtain TAC values at 0 s, 1 s,..., 280 s to cover the whole scan period. See Fig. 1 for an example.

**Fig. 2:**
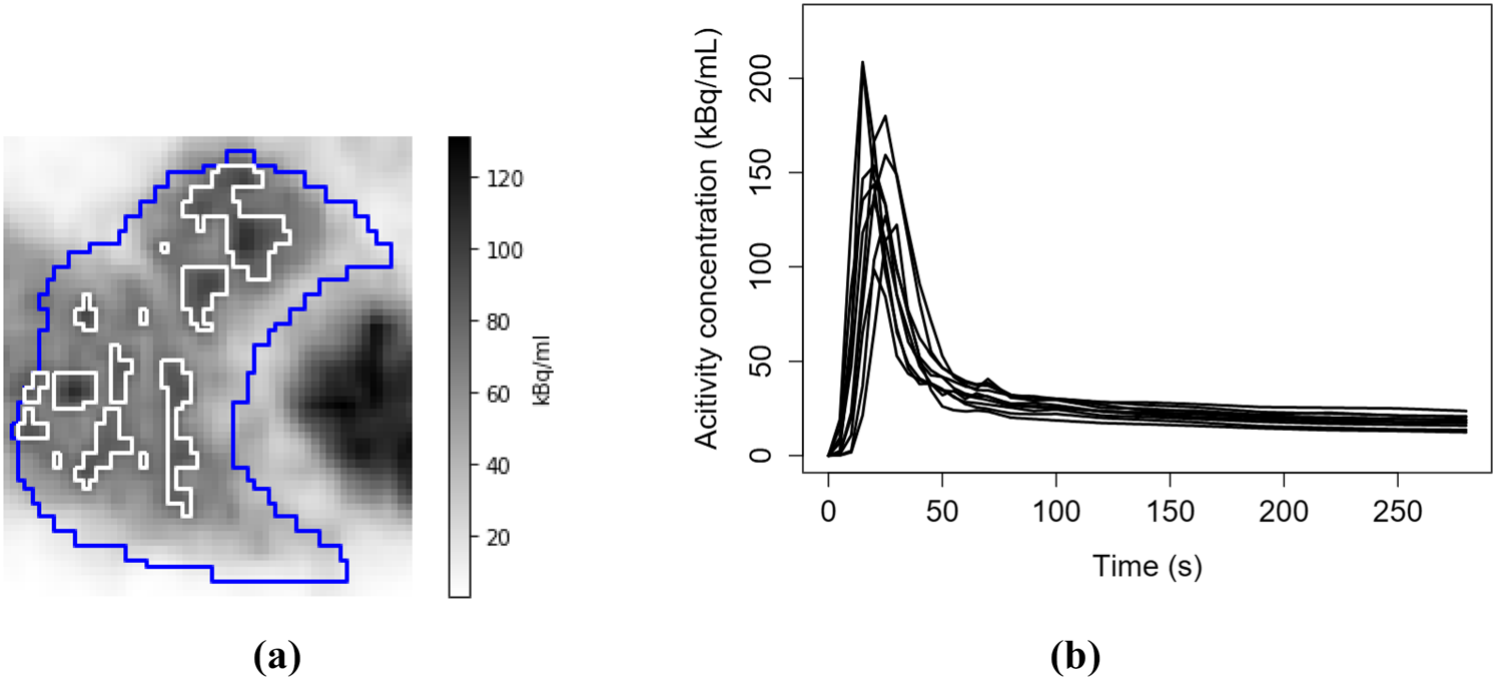
**a** The outlines of the original RV segmentation mask in blue and the outlines of the segments formed by the voxels over the 90th percentile in white in one cropped coronal slice of a PET image, and **b** the RV inputs of ten patients when computed as a mean TAC of the voxels over the 90th percentile

To derive the input function from the RV, we computed the mean TAC only from the RV voxels over the 90th percentile at the given time frame. The reason for this choice was that the input function is supposed to be the tracer concentration in the pulmonary blood inside the RV cavity and the whole RV segment also contains the ventricular walls and, due to the cardiac motion, possibly areas outside the heart, which would have lower tracer concentration than the actual pulmonary blood. After similar linear interpolation as to the lung TACs, we then visually checked the input TACs. As can be seen from Fig. 2, the resulting input functions have a clear peak and little noise, meaning that they are suitable for compartmental modelling [14].

### Compartment model

First, let us define a function $C_T(t)$ to express the tracer concentration at the lung tissue at the moment *t*. Similarly, let $C_B(t)$ be the tracer concentration in the pulmonary blood as a function of the time. The simple one-tissue compartment model (1TCM) with $C_B(t)$ as the input function can be described with the differential equation

1 $$\begin{aligned} \frac{\partial }{\partial t}C_T(t)=K_1C_B(t)-k_2C_T(t), \end{aligned}$$

where $K_1$ and $k_2$ are rate constants independent of time. This equation can be solved as

2 $$\begin{aligned} C_T(t)=K_1C_B(t)\otimes e^{-k_2t}\equiv K_1e^{-K_1t}\int ^t_0C_B(u)e^{k_2u}\,\textrm{d}u. \end{aligned}$$

Here, the rate constant $K_1$ expresses how much of the tracer in the pulmonary blood is delivered to the lung tissue, which is how we estimate of the PBF. It has non-negative values originally with the unit mL (of blood)/s/cm$^3$ (of tissue) in the model, but we convert them into mL/min/cm$^3$. The other rate constant $k_2$ is the blood flow exiting the lung tissue, which has an initial unit /s similarly converted later into /min. The ratio $K_1/k_2$ of rate constants, known as the water partition coefficient, shows how great percentage of the water stays in the tissue. Unlike Matsunaga et al. [9], we do not use the decay coefficient $\lambda$ in the model because our PET data is already corrected for decay.

However, it must be taken into account that the tracer concentration computed from our lung segments in the PET image is not directly comparable to the tissue concentration $C_T(t)$ above. Namely, while we removed the subclavian arteries and the brachiocephalic veins and the majority of the air, there is still air and other blood vessels left in the VOI selection. Consequently, we define another function $C_\textrm{PET}(t)$ that contains the tracer concentrations in the air near to lung lobe surface and in the blood within the remaining pulmonary arteries and veins in addition to the actual lung parenchyma tissue:

3 $$\begin{aligned} C_{PET}(t)=V_AC_A(t)+V_BC_B(t)+\left( 1-V_A-V_B\right) C_T(t), \end{aligned}$$

where $C_A(t)$ is the tracer concentration in the air with respect to the time and $V_A$ and $V_B$ are the volume fractions of the air and the blood, respectively. The volume fractions $V_A$ and $V_B$ are between 0 and 1 and do not have units. We assume that $C_A(t)=0$ as in [9], which results in the model

4 $$\begin{aligned} C_{PET}(t)=V_BC_B(t)+\left( 1-V_A-V_B\right) C_T(t), \end{aligned}$$

Furthermore, to account for the delay caused by the time it takes from the tracer to travel from the RV to the lung lobe of interest, we add an integer parameter *j* to the model. The possible values of *j* are 0,1,...,15 (unit: s) and *j* is included by simply replacing $C_B(t)$ in (1) by $C_B(t-j)$, similar to the research by Wang et al. [15].

### Model fitting

For any values $K_1,k_2\ge 0$, $V_A,V_B\in (0,1)$, and $j=0,1,...,15$, we found the fitted model TAC as follows: We assume that $C_B(0)=C_T(0)=0$ and, if $j\ge 1$, we extrapolate $C_B(-i)=0$ for $i=1,...,j$. By (1), we compute the values of the function $C_T(t)$ iteratively as

5 $$\begin{aligned} C_T(i)=K_1C_B(i-j-1)+(1-k_2)C_T(i-1), \end{aligned}$$

for each $n=280$ time points of $i=1,2,...,n$ s. We define $C_\textrm{PET}(t)$ as in (4). Finally, we compute the model error as the sum of the squared differences

6 $$\begin{aligned} \sum ^n_{i=1}\left( \tilde{C}(i)-C_\textrm{PET}(i)\right) ^2, \end{aligned}$$

where $\tilde{C}(t)$ is the measured mean TAC from the lung lobes. By minimizing this error term (6) with the non-linear Newton-type minimization algorithm nlm in R, we choose the values of $K_1,k_2,V_A,V_B$ for every $j=0,1,...,15$. We use initial values $K_1=k_2=1$ and $V_A=V_B=0.1$. Since nlm does not allow upper or lower bounds, we force $K_1$ and $k_2$ to be non-negative and $V_A$ and $V_B$ to be on (0, 1) with absolute value and the inverse-logit function. After the optimization, we fix the value of *j* by choosing the one with the lowest error term (6).

### Statistical testing

We reported the mean, standard deviation, and median values of the different model parameters. To estimate whether there are significant differences in the PBF values between different patient subgroups, we used the Mann–Whitney *U* test with a level of significance of 5%. We also computed Spearman’s correlation coefficients to detect possible association between PBF and other numerical variables collected from the patients.

## Results

Table 1 contains the mean and standard deviation values of all the parameters obtained by fitting the model to the mean TACs of different lung lobes and the mean TAC of all the five lung lobes. We see that the estimated PBF is lowest for the RLL and highest for the RUL.

**Table 1:** Mean ± standard deviation values of the fitted model parameters $K_1,k_2,V_A,V_B,j$ and the ratio $K_1/k_2$ for the mean TACs of different lung lobes and all of them computed from 103 patients

Lung lobe	$K_1$ (mL/min/cm$^3$)	$k_2$ (/min)	$K_1/k_2$	$V_A$ (%)	$V_B$ (%)	Delay *j* (s)
LLL	1.09±0.655	4.15±2.51	0.263±0.0517	11.0±1.54	1.31±1.48	0.447±0.967
RLL	0.978±0.845	4.05±3.91	0.256±0.0520	11.0±0.909	3.14±2.57	0.534±1.58
RML	1.90±1.90	10.9±12.3	0.179±0.0406	12.6±6.95	2.07±3.12	0.981±1.67
LUL	1.54±0.885	8.81±7.66	0.186±0.0410	11.2±1.14	1.85±2.56	0.874±1.46
RUL	2.66±2.11	15.0±12.6	0.183±0.0417	11.0±0.585	3.22±3.40	1.83±1.97
All lobes	1.21±0.825	5.85±4.06	0.207±0.0358	11.0±0.696	2.90±2.93	0.437±1.12

**Table 2:** Median values of the fitted model parameters $K_1,k_2,V_A,V_B,j$ and the ratio $K_1/k_2$ for the mean TACs computed from mean TACs of all five lung lobes over the specified subgroups of *N* patients within the 103 patients of Table 1

Patient group	*N*	$K_1$	$k_2$	$K_1/k_2$	$V_A$ (%)	$V_B$ (%)	Delay *j*
		(mL/min/cm$^3$)	(/min)				(s)
Women under 65	23	1.41	5.84	0.218	10.9	2.73	0
Women over 65	33	1.06	5.45	0.200	10.8	2.51	0
Men under 65	27	1.03	5.01	0.205	10.9	1.53	0
Men over 65	20	0.780	3.91	0.198	11.0	1.16	0
All women	56	1.24	5.55	0.205	10.9	2.62	0
All men	47	0.842	4.69	0.205	11.0	1.50	0
All patients under 65	50	1.22	5.56	0.213	10.9	2.21	0
All patients over 65	53	1.01	4.94	0.199	10.9	2.23	0
All patients	103	1.03	5.04	0.205	10.9	2.23	0

**Table 3:** The *p*-values of Mann–Whitney *U* test comparing the estimated PBF values (equal to $K_1$ values) prior to any correction for multiple comparisons between the subgroups of Table 2

1st group	2nd group	*p*-value
Women under 65	Women over 65	0.498
Men under 65	Men over 65	0.709
Patients under 65	Patients over 65	0.695
Women under 65	Men under 65	0.151
Women over 65	Men over 65	0.312
Women under 65	Men over 65	0.123
Women	Men	0.0882

None of the *p*-values are statistically significant

The patients were then divided into different subgroups based on their age and sex and the PBF values were tested for statistically significant differences between the subgroups. The median values of the parameters are in Table 2 and the related *p*-values in Table 3. According to these tables, PBF was lower for men and patients over 65 years but not statistically significantly so.

**Table 4:** Spearman’s correlation coefficients between the estimated PBF values (equal to $K_1$ values) and the age, the weight, and the BMI among the women, the men, and all the 103 patients

	Women (*N*=56)	Men (*N*=47)	Patients ($N=103$)
Age	$-$0.0558	$-$0.149	$-$0.0618
Weight	0.102	0.0583	$-$0.00925
BMI	0.169	0.103	0.135

None of the correlation coefficients are statistically significant according to a *t*-test for correlation

**Fig. 3:**
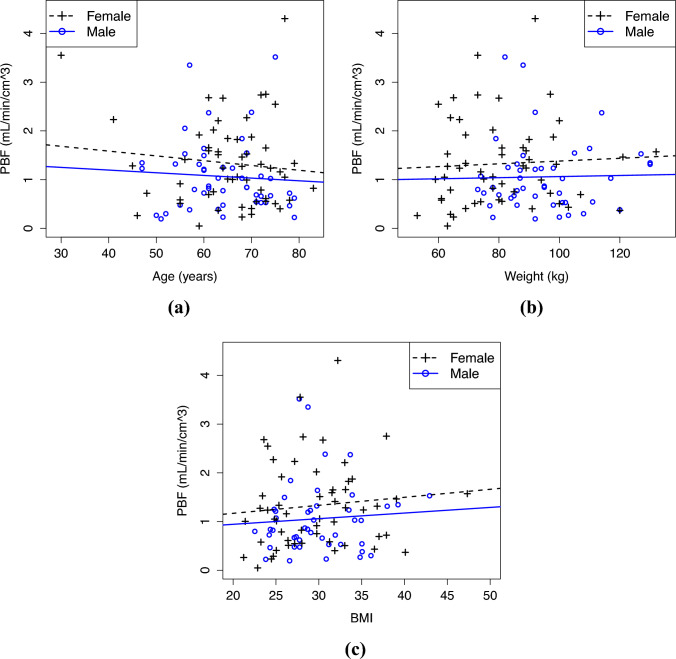
The estimated PBF values (equal to $K_1$ values) computed from the mean TACs of all five lung lobes against **a** the age, **b** the weight, and **c** the BMI of all the 103 patients. The least squares regression lines were computed separately for men and women. The related Spearman’s correlation coefficients are in Table 4

Figure 3 has the PBF values plotted against age, weight, and BMI with least squares regression lines and Table 4 has the related Spearman’s correlation coefficients. According to Table 4, there is no significant correlation. Figure 3 shows that the individual variation is very high, which hinders the detection of any association between the variables.

## Discussion

In this study, we computed PBF estimates for 103 patients based on their $^{15}$O-water PET images, resulting in a mean PBF of 1.21±0.825 mL/min/cm$^3$ and a median PBF of 1.06 mL/min/cm$^3$ over all the lung lobes. These are in harmony with the earlier study by Schuster et al. [8], who reported a mean PBF of 1.21±0.32 mL/min/cm$^3$ and 0.57±0.33 mL/min/cm$^3$ for 15 normal subjects and five cardiomyopathy patients, respectively. However, in the research by Matsunaga et al. [9], the mean PBF estimates varied from 1.4±0.3 to 5.3±0.6 mL/min/cm$^3$ for the same nine lung cancer patients, depending on the choice of the model. Since there are only two other studies and the number of subjects is quite limited in both them, it is difficult to estimate what kind of PBF values would be realistic with this method.

However, it was noted by research by Matsunaga et al. [9] that while the PBF estimates varied much between the models, the water partition coefficient, equal to the ratio $K_1/k_2$ here, had mean values of 0.17±0.03 and 0.60±0.08. We obtained a mean value of 0.207±0.0358 for $K_1/k_2$ with a very similar model than the one resulting in the value of 0.17±0.03 by Matsunaga et al., suggesting that this ratio might be more trustworthy quantity used to compare the patients. According to our results, the values of $V_A$ also consistently stayed close to 11%, which could correspond with the amount of the air left in the hollow lung lobe VOIs or be caused by potential over-parametrization in the model. However, we cannot compare $V_A$ or the other non-flow model parameters because their values were reported neither by Matsunaga et al. nor by Schuster et al. [8].

To evaluate this potential over-parametrization in more detail, we computed Aikaike’s information criteria (AIC) from the current model as in [16], the version of the model without the parameter $V_A$, and the version with neither $V_A$ nor $V_B$. Based on the median AICs, the best model was the one with $V_B$ but not $V_A$. This leads to a question about the appropriate selection of the parameter $V_A$. Namely, even though the use of this parameter causes over-parametrization in our model, there is still some air left in the lung lobe VOIs that should be accounted. In the earlier study, Matsunaga et al. [9] used an external $^{137}$Cs point source to obtain transmission images and fixed the value of $V_A$ according to a voxel-based tissue fraction computed from the transmission images.

We noted large differences in PBF estimates between different lung lobes: The mean PBF was 0.978±0.845 mL/min/cm$^3$ in RLL but nearly thrice as much in RUL. This is likely explained by the fact that there is large vessels left in the VOIs of the upper lung lobes besides the ones we removed. It is also possible that there is spill-over from the heart or some other organ for the upper lung lobes, especially due to the cardiac and respiratory motions. Namely, as TotalSegmentator only performs robust segmentation from CT image, it will not provide accurate segmentation on different time frames of the dynamic PET image. Additionally, our method of deriving hollow lung lobe VOIs to decrease the amount of air in the VOIs was also very robust.

Naturally, the values of $K_1$ and therefore the PBF estimates also strongly depend on the values of the input function. Schuster et al. [8] used the voxel with the peak value and Matsunaga et al. [9] plotted the RV volume of interest manually by including always the maximum voxel of consecutive image planes. We would obtain lower PBF estimates by using the maximum TAC of the RV VOI as opposed to our current choice of the mean of the TACs over the 90th percentile. However, the maximum TAC of the RV VOI would not give us the desired average tracer concentration in the pulmonary blood but its maximum instead. Additionally, this method would be very sensitive to error caused by, for instance, random coincides during the PET scan.

According to our results, the PBF is higher for women than for men and higher for younger patients than those over 65 years but not significantly so. In earlier research by Aanerud et al. [17], it has been noted that cerebral blood flow is considerably higher for young women than for young men but, because the cerebral blood flow decreases faster in women as they age, this difference will even out by the age of 65 years. Given our patients had a mean age of 64.8±9.33 years, it is therefore possible that there are significant differences in PBF between sexes but our population is too old to have them.

It should be taken into account that our study population was recruited among the patient showing chest pain and symptoms of a possible coronary artery disease. Consequently, we can only get a general view of what the PBF would be like in this type of population subgroup (middle-aged or older, high BMI, likely pre-existing heart conditions) as opposed to information about PBF in healthy humans. It is possible that some of the patients had other significant conditions affecting their PBF. While we used non-parametric statistical testing to avoid wrong conclusions caused by possible outliers, it is still possible that some of the differences noted in this study would not exists in different subject groups. Similarly, it is possible that there are some relationships present in younger people that we were not able to detect.

In future study, it would be important to also study this topic for younger people to find out if there are significant differences between sexes or a negative trend with PBF and age. One interesting topic for future research could be to design a multi-compartment model to account the blood flow between different lung lobes instead using the standard 1TCM. Additionally, our method could be studied further by investigating its reproducibility between several PET scans of the same patient. Furthermore, the association of PBF and blood pressure, heart rate, and respiratory rate could be investigated.

## Conclusion

In this work, we studied PBF quantification by systematically fitting the 1TCM to the $^{15}$O-water PET data from 103 patients with automatic VOI selection. We utilized the help of the automatic segmentation tool TotalSegmentator to locate the anatomic structures of interest and used binary erosion and dilation algorithms to produce hollow lung lobe VOIs without the subclavian arteries and the brachiocephalic veins. The estimated mean PBF was in harmony with the other two existing studies, though quite high in the upper lung lobes, likely due to the overestimation caused by the high tracer concentration in the blood within the pulmonary vessels. The female patients had higher PBF than the male and the younger patients higher PBF than the patients over 65 years but neither of these differences was statistically significant because of the very high individual variation between different patients. In future research, more careful segmentation might be required for upper lung lobes but our method still offers a very efficient way to obtain relatively reasonable PBF estimates. Additionally, more research is warranted on how to estimate the air volume around the lung lobe VOIs in a way that does not result in an over-parametrized model.

## Data Availability

The patient data are not available due to privacy restrictions.

## Code availability

Available at github.com/rklen/Pulmonary_blood_flow
